# Developing Concepts for Neuroscience: A Philosophical Toolkit

**DOI:** 10.1111/ejn.70403

**Published:** 2026-01-20

**Authors:** Philipp Haueis, Daniel S. Margulies

**Affiliations:** ^1^ Department of Philosophy Bielefeld University Bielefeld Germany; ^2^ Institute of Philosophy Leibniz University Hannover Hannover Germany; ^3^ Centre National de la Recherche Scientifique (CNRS) Integrative Neuroscience and Cognition Center Paris France; ^4^ Wellcome Centre for Integrative Neuroimaging University of Oxford, John Radcliffe Hospital Oxford UK

**Keywords:** conceptual development, cortical column, cortical gradient, default mode network, epistemic goals, hierarchy

## Abstract

Alongside models and methods, concepts are crucial tools to study and understand the brain. They help us pursue various goals, such as describing phenomena based on patterns in the data or explaining why these phenomena occur. Yet while terms such as “action potential” or “network” guide our efforts to reach these goals, other concepts have failed to advance our understanding of the brain. In this paper, we draw on recent work from philosophy of science to show that the success or failure of concepts in neuroscience depends on the epistemic goals the field aims to achieve. Looking at cases such as “default mode network,” “cortical column,” and “hierarchy,” we formulate conditions under which introducing, refining, or replacing a concept succeeds or fails. These cases suggest that to better evaluate our concepts, we should make explicit which goals we aim to achieve when using them.

## Graphical abstract

Whether and how concepts should be developed depends on the phenomena that neuroscientists aim to describe, classify, and explain. These epistemic goals shape when introducing novel terms like “default mode network,” refining existing terms such as “hierarchy,” and retiring or replacing outdated ones such as “cortical columns” succeeds or fails to advance our scientific understanding of how the brain works.

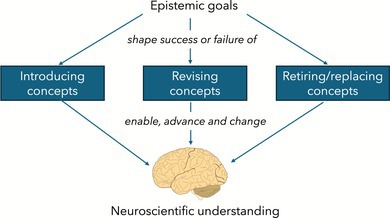

**Abbreviation**
DMN: default mode network

## 1 Introduction

Neuroscientists frequently discuss which methods and models to use when studying a given phenomenon (Bandettini 2009; Bassett et al. 2018). They less often ask which concepts are adequate to study the brain (Stern 2017). Yet, terms like “neuron,” “action potential,” “receptive field,” or “network” are part and parcel of neuroscientific practice. Without them we could not communicate the results of our studies and think about how the brain is organized. Neuroscientific concepts thus reflect our scientific understanding of the brain: We speak of neurons (rather than reticula), or networks (rather than isolated areas) because we think these are important units to describe, classify, explain, or predict what the brain does. Such conceptual developments are often intertwined with technological advances (Bickle et al. 2022). Staining methods were crucial for Cajal to develop the neuron doctrine (and his controversy with Golgi); single‐cell recording was key for forming concepts like “receptive field” or “feature detector” (Martin 1994; Chirimuuta and Gold 2009; Shepherd 2010); and connectivity analyses and graph theory are crucial for describing network organization of the brain (Kötter and Stephan 2003; Sporns 2011).

While many would agree that the above concepts advanced our understanding of the brain, not every conceptual innovation achieves this effect. For example, while the term “grandmother cell” captured neuroscientists' thinking about hierarchical coding, it could not be empirically substantiated and fell out of fashion, giving way to alternative ideas like sparse coding (Barwich 2019). And while “cortical column” was part of research that won Hubel and Wiesel the Nobel Prize, critics recently questioned the functional relevance of these vertical cell bands in the cortex (Horton and Adams 2005; Haueis 2021). These examples show that concepts and conceptual development can *succeed* or *fail* to advance our scientific understanding of the brain. But what distinguishes successful from failed conceptual development in neuroscience?

In this paper, we show that philosophical work on conceptual development provides useful tools to answer this question. Our main thesis is that one cannot talk about the success or failure of concepts in neuroscience without looking at the *goals* a field aims to achieve. Our thesis follows philosophers of science who adopt a pragmatist epistemology to argue that scientific concepts are like tools (Feest 2010, 2025). Like material tools, they can be fit for certain purposes but not others, and scientists modify them to get closer to a goal (Steinle and Feest 2012). In scientific practice, the goals are *epistemic—*they are related to knowledge of the phenomena that a field studies (Brigandt 2010). Exemplary types of epistemic goals are *describing* phenomena based on patterns in the data, *classifying* phenomena into different kinds, *explaining* why they occur, or *predicting* their occurrence.

We will use the notion of epistemic goals as a heuristic guide to identify putative cases of successful or failed concepts in neuroscience. We then use these cases to extract general conditions for when introducing, revising or replacing a neuroscientific concept succeeds or fails to contribute to epistemic goals. This will provide support for our second thesis, namely, that successful concepts enable a better understanding of the brain, because they allow us to better describe, classify, explain, or predict important phenomena in the brain. The main takeaway of our paper is thus that success or failure of conceptual development is not absolute but always depends on the goals scientists want to achieve when using the concepts. It is thus crucial to make these goals explicit.

The paper is organized as follows: Section 2 discusses the case of “default mode network” to ask when introducing concepts to neuroscience research succeeds or fails. We identify the epistemic goals to which this particular concept contributes and specify general conditions under which introducing a concept contributes to epistemic goals in neuroscientific practice. In Section 3, we use “cortical column” as a putative failure case, specify its epistemic goals and general conditions under which researchers should “retire” or replace a concept. We show that the notion of an epistemic goal is helpful to distinguish between the need to retire and replace an old neuroscientific concept. In Section 4, we refine our view and argue for our second thesis that successful concepts enable a better understanding of the brain. We look at various interpretations of “hierarchy” in neuroscience to illustrate our case. Section 5 concludes.

## 2 Introducing Novel Concepts in Neuroscientific Research

During neuroscientific discovery, we frequently encounter previously unknown patterns of experimental data or model results. The goal of a scientific field then is to characterize these patterns adequately. Achieving this goal often requires introducing a novel concept (Steinle 2016). A familiar example is Alexander Fleming's discovery of penicillin. After discovering an unexpected pattern of bacterial growth in a petri dish, Fleming isolated the mold from this region and named the active entity preventing growth “penicillin” (Gaynes 2017). A similar neuroscientific example of concept development is “cortical column,” which Vernon Mountcastle introduced after discovering a vertical pattern of functional responses in the cat somatosensory cortex (see further discussion in Section 3).

The two cases above illustrate the value of stepping outside traditional hypothesis‐driven research for introducing novel concepts. One such kind of research is exploratory experimentation (Hubel and Wiesel 1998; Hussain and Cohen 2017; Colaço 2018; Haueis 2023).^2^ When the accepted conceptual frameworks are insufficient to characterize newly discovered phenomena, exploratory experiments can help researchers to form novel concepts to understand them (Steinle 2016; Feest 2025). Importantly, the need to introduce novel concepts can range from characterizing chance discoveries (such as in Fleming's case) to more systematic modes of exploration (such as in Mountcastle's case).

### 2.1 When Does Introducing Concepts in Neuroscience Succeed or Fail?

When does a concept help researchers in achieving the goal of adequately characterizing an unexpected pattern? Following Haueis (2023), we propose that a newly introduced concept is successful when it fulfils two conditions. The first condition is that it should be *anchored in a property* that is significant for pursuing an epistemic goal. In other words, we want the concept to capture features of the world that are important for our scientific projects.^2^ For example, in the case of Fleming, his epistemic goal was to understand the conditions under which bacterial growth occurred. His accidental discovery—and subsequent concept formation of “penicillin”—contributed to this goal by specifying when bacterial growth was inhibited. The second condition is that the novel concept should be *open‐ended*—i.e., applicable to novel contexts. This is important because, for a concept to initiate change, other researchers should be able to apply the novel concept in subsequent studies.

Let us look at the first condition—that of being anchored—in more detail. There are three general steps involved in identifying an anchoring property in exploratory experiments. The first step is *operationalization*—making the concept measurable in an experiment. Operationalization is successful depending on whether we choose adequate experimental conditions to observe the property that should anchor our concept. The second step concerns *significance*—assessing whether the operationalization has some form of external validity. We can usually infer significance by comparing experimental conditions to real‐world conditions under which we expect the anchoring property to occur. The third step concerns *reference*—checking whether the instrument we used is sufficiently precise to identify the property that we measured (Step 1) and assessed as functionally significant (Step 2).

When are novel concepts open‐ended? First, we need to ensure that other researchers can apply the concept beyond the context in which it was formed. One way to do this is to show that the concept is an instance of a more general concept. Second, the concept needs to be adaptable to the empirical details that differ from the context of its introduction. One way a concept is adaptable is when it can be applied under a variety of experimental conditions or involve different experimental or modeling techniques. Consequently, concepts can fail to be adaptable when their operational definition is tied to particular conditions and techniques, which constrains their open‐endedness.

### 2.2 Example: How Successful Was the Introduction of “Default Mode Network”?

In practice, the introduction of a novel concept should serve specific epistemic goals by both anchoring the concept in a property and ensuring its open‐endedness for broader application. The case of the “default mode network” (DMN) is a compelling example of this balance. Its introduction emerged from the convergence of two investigative pathways—one from neurophysics (Biswal et al. 1995) and the other from cognitive neuroscience (Shulman et al. 1997)—a convergence detailed elsewhere (Callard and Margulies 2011; Biswal 2012; Raichle 2015). Greicius et al. (2003) demonstrated that the areas showing deactivation during goal‐directed tasks (as highlighted by the cognitive neuroscience approach) are also “functionally connected,” exhibiting correlated fluctuations in activity at rest (as identified by the neurophysics approach). How successful was the introduction of the “default mode network”?

To answer this question, we must first specify the epistemic goals of these converging fields—neurophysics and cognitive neuroscience. In cognitive neuroscience, a key aim is to understand how brain activity relates to behavior and cognitive function (Bechtel 2008). In contrast, in neurophysics, a key aim is to relate the measured signal to organizational features of the brain (Buxton 2013). The use of low frequency fMRI oscillations when applying “default mode network” contributes to the neurophysics goal. The signal approximates anatomically bounded brain systems (e.g., Glasser et al. 2016), and in part reflects functional processes that maintain the integrity of these systems across global brain states like wakeful rest or sleep (e.g., Larson‐Prior et al. 2009). But does “default mode network” also identify an anchoring property that contributes to the goals of cognitive neuroscience, by relating DMN activity to behaviorally relevant information processing?

**FIGURE 1:**
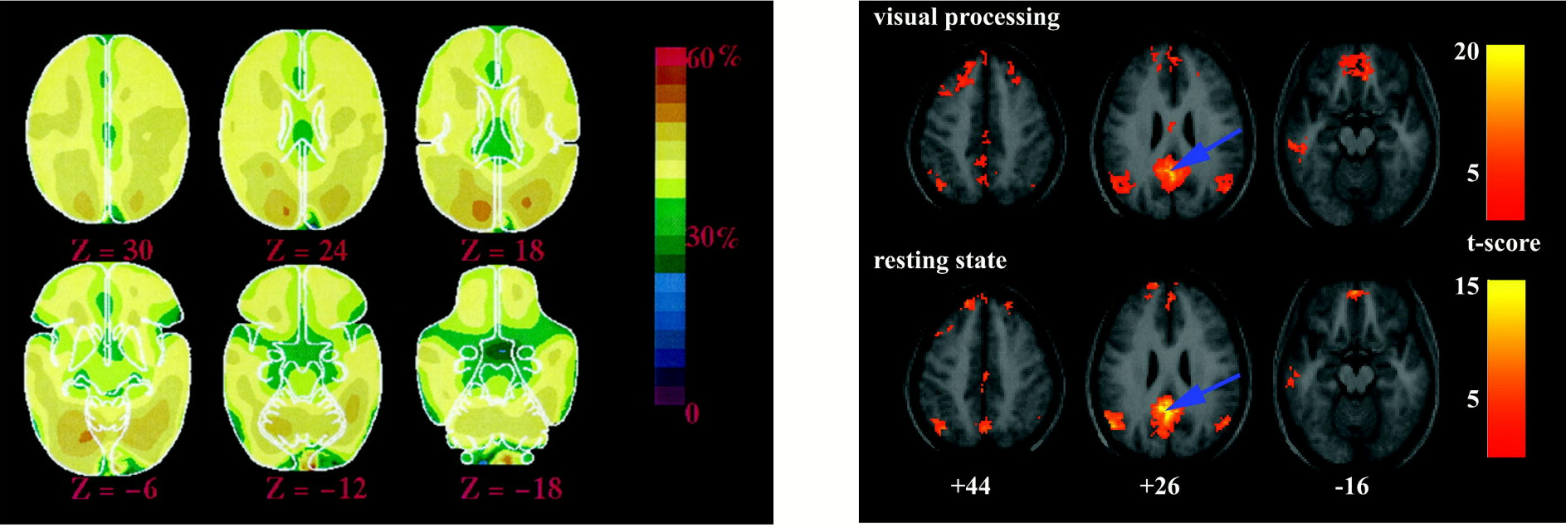
Left: PET images showing that percentage of available oxygen delivered is relatively constant across the brain during the resting state (subject is lying awake in the scanner, eyes closed). *Source:* Adapted from Raichle et al. (2001). Right: fMRI images showing that posterior cingulate cortex is functionally connected to the same areas during when subjects view a fixation cross (upper row) and during the resting state with eyes closed (lower row) (Adapted from Greicius et al. (2003), Figure 3).

Based on our previous work (Haueis 2023), we argue that “default mode network” fails to identify an anchoring property for the goals of cognitive neuroscience, because the property that it identifies fails to relate DMN activity to cognitive brain function. Specifically, the “default mode” in the “default mode network” was intended to capture a unique neural signature—operationalized as a uniform oxygen extraction fraction that reflects an equilibrium between blood flow and oxygen uptake (Raichle et al. 2001). However, this operational definition does not clearly distinguish the specific group of regions identified in subsequent studies (e.g., Greicius et al. 2003) from the rest of the brain (Figure 1, Klein 2014).

Rather than highlighting a distinct cognitive functional property, the metabolic measure suggests a “default mode [that] is characteristic of all brain areas at all times” (Raichle and Snyder 2007). “Default mode” thereby fails to capture the unique feature that sets apart the *default mode network regions* that deactivate during goal‐directed tasks. These areas, identified by Shulman and colleagues, are significant for cognitive neuroscience precisely because their deactivation implies a state of sustained information processing at rest (Gusnard and Raichle 2001)—a phenomenon that the metabolic definition of “default mode” does not adequately address. The default mode concept does not capture this sense of functional activity because it describes the brain in terms of metabolism and flow of energy, and not in terms of cognition and information processing. Thus, while “default mode” is anchored in a *metabolic* property, “default mode network” fails to relate DMN activity to *cognitive* brain function.

The example of introducing “default mode network” illustrates why a lack of anchoring properties does not stand in the way of a concept being open‐ended. “Default mode network” is open‐ended because it identifies a network that can be investigated under a wide variety of experimental conditions, using different experimental techniques. This network can be detected in task conditions related to different cognitive functions (e.g., mind‐wandering, autobiographical memory, internally oriented attention) and many different neurological and mental disorders (e.g., Alzheimer's, autism, anxiety, depression, and schizophrenia, Buckner et al. 2008, Broyd et al. 2009, Smallwood et al. 2021). By contrast “default mode” is much less open‐ended, because its operational definition is limited to PET imaging recordings (Raichle and Snyder 2007).

The above analysis suggests that the success of introducing “default mode network” was mixed, since Greicius et al.'s introduction of the concept, while open‐ended, failed to identify an anchoring property for the goals of cognitive neuroscience. This leaves us with a conundrum, while our philosophical evaluation suggests a mixed success, neuroscientists widely consider that the discovery of DMN changed our understanding of the brain significantly (Callard and Margulies 2014; Raichle 2015). What explains this discrepancy?

We propose that this discrepancy can be explained by the *strategic ambiguity* inherent in the term “default mode network.” We already noted that the use of “functional connectivity” to identify the DMN is ambiguous. It can mean a noncognitive reading of “function” as brainwide maintenance and repair of functional systems (Haueis 2018) or a cognitive reading of “function” and endogenous information processing within these systems that supports behavior (Bechtel 2013). But even for the goal of describing the system's cognitive functions, “default mode network” is strategically ambiguous. On the one hand, “default mode” connects the Greicius study to the cognitive neuroscience pathway in which researchers discovered that these areas engage in sustained information processing during the experimental resting state (Raichle et al. 2001). On the other hand, the term is neutral between different cognitive hypotheses, e.g., that these areas support processing tasks related to the “self” (Gusnard and Raichle 2001) or that they are critical for retrieving episodic memories (Greicius et al. 2003). In the initial stages of its conceptual development, “default mode network” marks the epistemic uncertainty regarding which of these hypotheses is correct. The term thus encourages researchers to pursue multiple different hypotheses rather than prematurely closing off alternatives. This suggests that when a study fails to identify an anchoring property, a strategically ambiguous term actually increases open‐endedness. This value of using ambiguous terms to reflect imprecise concepts is known from other disciplines, such as “gene” in molecular biology (Rheinberger and Müller‐Wille 2018).

While strategic ambiguity explains how the term “default mode network” signals uncertainty, the underlying concept should eventually identify an anchoring property relevant to one's epistemic goals.^3^ One such proposal comes from our own work (Margulies et al. 2016), where we sought to refine the concept by situating the DMN along a principal axis of *representational abstraction* that distinguishes primary sensory areas from transmodal regions. This formulation tentatively identifies an anchoring property to describe cognitive function—namely, the DMN's role in processing information that is least directly tied to sensory input. The DMN could thereby be characterized by processing at the highest degree of representational abstraction. This constitutes an anchoring property for the goals of cognitive neuroscience because it makes a claim regarding the characteristic cognitive profile of the DMN. Anchoring “default mode network” in this property contributes to the epistemic goal of describing behaviorally relevant information processing in the brain.

The case of the DMN illustrates that a concept's success cannot be judged solely by its initial empirical anchoring. Strategic ambiguity, far from being a weakness, can be a valuable feature that maintains a concept's open‐endedness and stimulates further investigation (Sterner 2022). This example underscores the importance of explicitly considering epistemic goals in conceptual development; even when a concept fails to fully satisfy all conditions at its inception, its capacity to foster ongoing and broad inquiry may ultimately contribute to a deeper understanding of brain organization.

## 3 Retiring or Replacing Old Concepts: The Case of “Cortical Column”

While we just saw when novel concepts help or hinder us to pursue research goals, we now turn to the question when already established concepts fail to be useful in this regard. In ongoing research, the goals of inquiry are usually implicit and the tools used to pursue them are imperfect; both only become clearer once a research program develops (Bechtel and Richardson 2010; Feest 2025). It can therefore happen that a scientific concept, even though initially deemed helpful for solving a particular problem, becomes outdated and unhelpful to pursue the goals of the research community. In this situation, we may want to stop using the outdated concept or even replace it with an alternative.

### 3.1 When Should We Retire or Replace Old Concepts?

**FIGURE 2:**
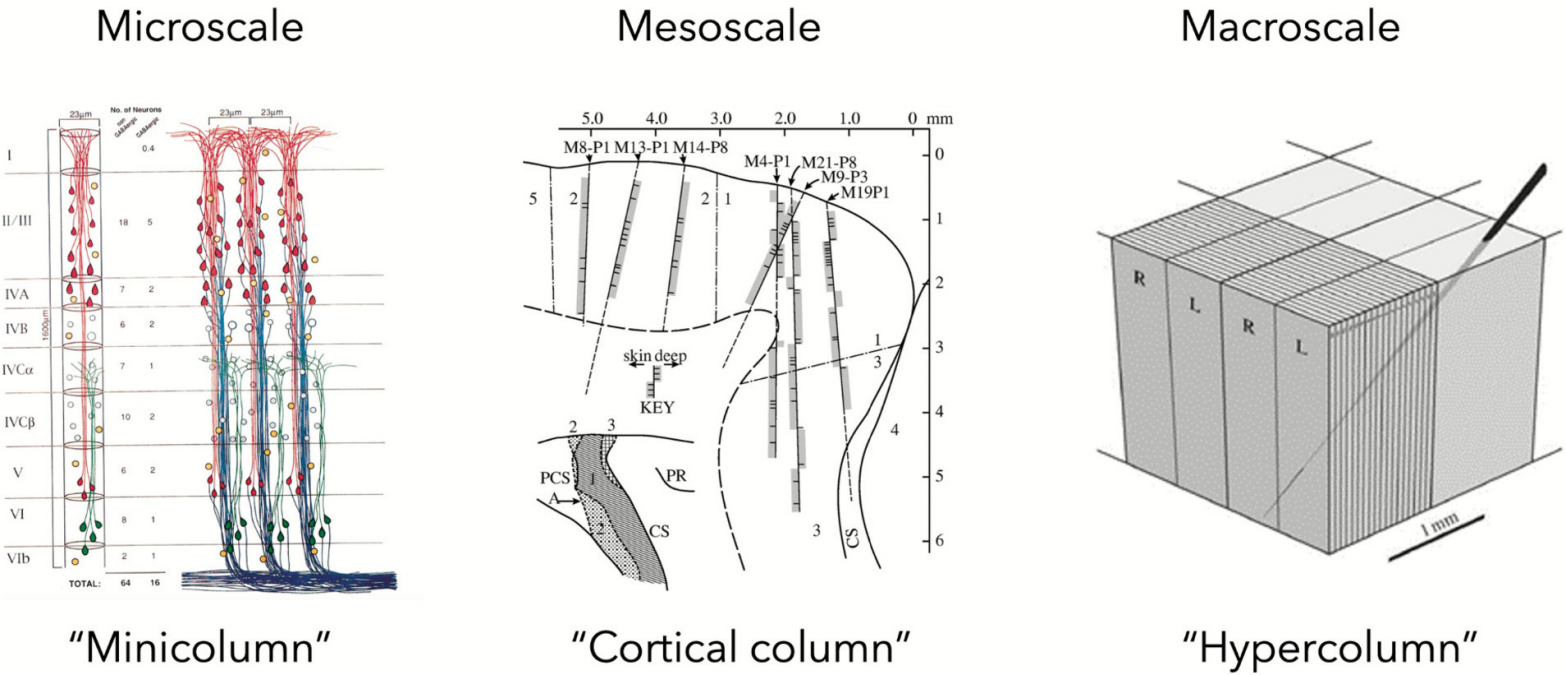
Different uses of the cortical column concept. At the microscale, “minicolumn” targets vertical connections of cell bands within a 30‐μm radius (Left, adapted from Mountcastle 1997). At the mesoscale, “cortical column” targets functionally modular responses to stimuli in vertical electrode recordings (Here: Modality‐specific responses in macaque primary somatosensory cortex, adapted from Powell and Mountcastle 1959). At the macroscale, “hypercolumn” targets multiple columnar system, which are arranged in a regular fashion (Here: Sequence‐regular orientation columns intersect orthogonally with alternating left and right ocular dominance columns, this organization is reiterated across entire V1, image from Horton and Adams 2005).

An example of a putatively outdated concept in neuroscience is “cortical column,” which refers to vertical structures whose neurons show similar responses to a sensory stimulus. In the mid‐1950s, Mountcastle introduced this term to relate the physiological response patterns he recorded in cat somatosensory cortex to radial cell bands seen in histological sections (Mountcastle 1957). Figure 2 shows how “cortical column” was subsequently extended to new areas and scales of cortical organization. While it enabled Nobel Prize winning research by Hubel and Wiesel (1977), some researchers also criticized “cortical column” as referring to a structure without a function (Horton and Adams 2005), called to retire it from current research (Haueis 2021), or even replace it with alternatives like “canonical microcircuit” (da Costa and Martin 2010). In this section, we take this putative example of conceptual failure to explicate the conditions under which neuroscientists should stop using an outdated concept or replace it with an alternative.

To answer whether we should stop using or replace “cortical column,” we first need to ask what distinguishes these options. Like with introducing novel concepts, we argue that retiring or replacing outdated concepts depends on the *epistemic goals* the community pursues. While a community will usually pursue multiple goals when using a concept, there is often a *central* epistemic goal to which it should contribute. For example, the central goal of using “gene” is to understand—i.e., describe, classify, predict, and explain—phenomena of inheritance (Rheinberger and Müller‐Wille 2018). Whatever else biologists use it for, it would be a problem for biology if “gene” did not contribute to a better understanding of inheritance. Similarly, the central epistemic goal of “cortical column” in neuroscience is to *identify a building block of the mammalian neocortex*, similar to how “neuron” or “cortical area” contribute to the goal of finding building blocks at different scales. In the case of “cortical column,” the building block is supposed to be an anatomically discrete functional module that performs the same computation everywhere across neocortex (Mountcastle 1997). Proponents and critics agree that whatever else “cortical column” is useful for, this is the central goal of using the column concept in physiology and comparative neuroanatomy (Mountcastle 1978, 1997; Swindale 1990; Purves et al. 1992; Catania 2002; Horton and Adams 2005).

Based on previous work, we propose that a concept should be retired from research if it *persistently fails to contribute to its central epistemic goal* (Haueis 2021). The failure needs to be persistent because scientific concepts, which are tied to empirical data, are never perfect and are always capable of evolving. We cannot simply point to current empirical shortcomings or incomplete explanations and proclaim that a concept is outdated. Future research may solve these issues. This suggests that outdated concepts are plagued by problems that persist, even after improving methods or reinterpreting key assumptions. Furthermore, the central epistemic goal is important to see that the various persistent problems of a concept are not disconnected failures. They must all hinder us from achieving the central epistemic goal.

The central epistemic goal also distinguishes retiring a concept from replacing it with an alternative. First, it only makes sense to replace a concept if the community thinks that the central goal is still worth pursuing. Sometimes we come to think that a research question is itself ill‐posed, and that trying to answer it does not help us better understand the relevant phenomena. For example, Horton and Adams (2005) deny that we should search for a common building block because “unravelling the organization of the cerebral cortex will require a painstaking description of the circuits, projections and response properties peculiar to cells in each of its various areas.” By contrast, researchers who want to replace “cortical column” continue pursuing its central goal of identifying a common building block (da Costa and Martin 2010).

Second, a replacement concept needs to avoid the persistent problems that hindered researchers using the old concept to achieve the central epistemic goal. History of science teaches us that not every call for replacement succeeds. While biologists successfully replaced “germ plasm” with “gene” to describe the unit of inheritance, calls to replace “gene” with “cistron,” “recon” and “muton” failed to catch on (Rheinberger and Müller‐Wille 2018). The condition to avoid problems is important because otherwise, our replacement efforts become relabeling exercises that fail to address the underlying issues which made the old concept outdated in the first place.

### 3.2 Example: Retiring and Replacing “Cortical Column”

**FIGURE 3:**
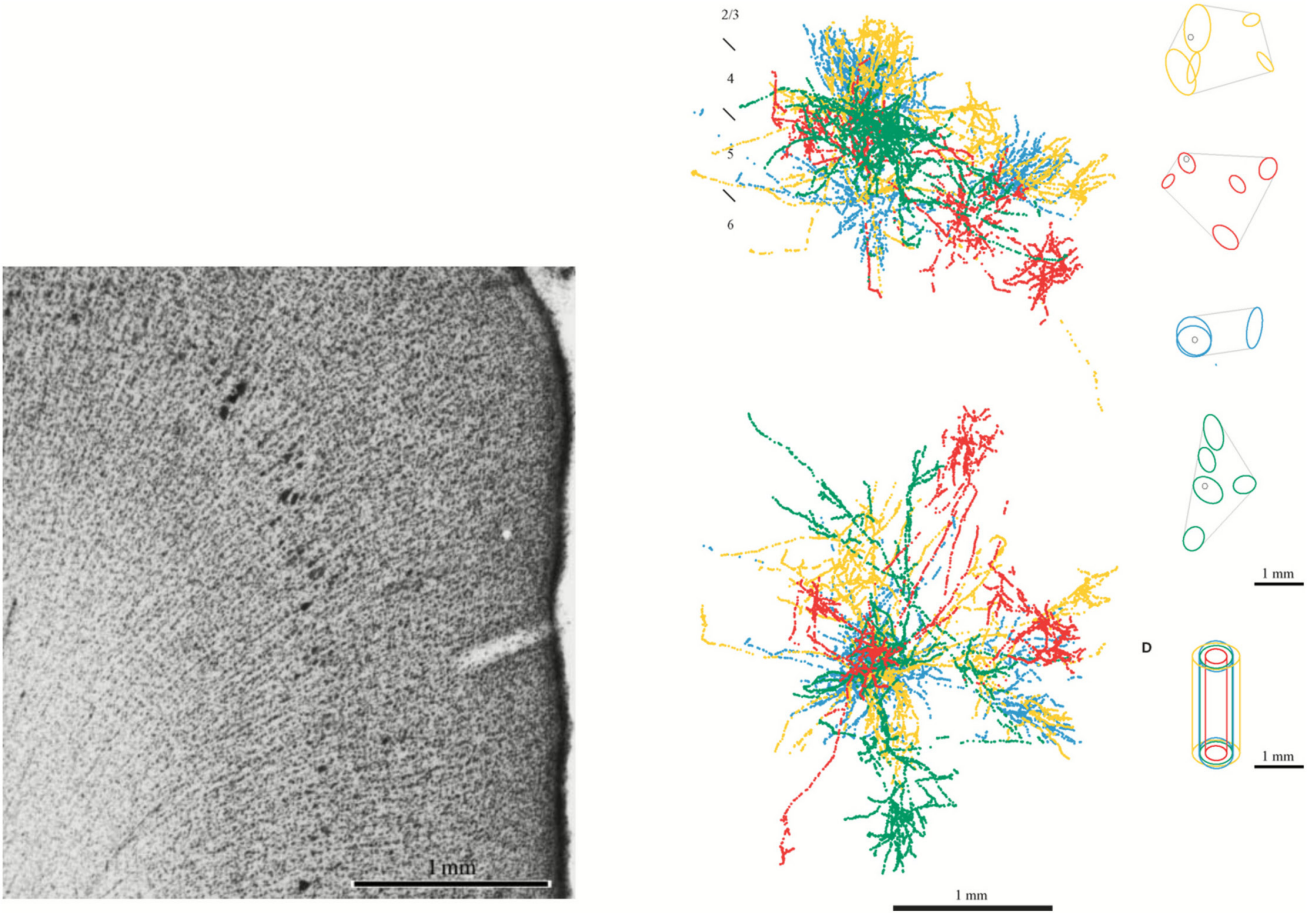
The problem of missing columnar boundaries. *Left:* Nissl‐stained cross section of macaque somatosensory cortex shows vertical cell bands throughout all cortical layers, but no discernable boundaries between them (Adapted from Horton and Adams 2005). *Right:* Stereotypical bouton clustering of neurons in different layers of cat V1 (yellow: L2/3 pyramidal cell, red: L4 spiny stellate cell, blue: L5 pyramidal green: L6 pyramidal cell). In all cases axons extend beyond multiple minicolumns (Adapted from da Costa and Martin 2010).

To answer whether we should retire or replace “cortical column,” let us start by looking at three problems that affected the search for a basic building block in the cortex. First, the *problem of missing boundaries* was already known to Mountcastle (1957): while physiological responses shift abruptly every 500 μm, Nissl‐stained sections show no discrete anatomical boundaries that would separate one column from another (Figure 3 left). To solve this issue, Mountcastle (1978) introduced “minicolumn” and proposed that vertical cell bands at the microscale are separated because their vertical connections are stronger than their horizontal connections (Figure 2a). While this solution was initially empirically supported (Peters and Sethares 1996), improved methods showed that the problem of missing boundaries persisted. In Figure 3 (right), 90% of synapses connect with neurons further than 100 μm away from the minicolumn.

**FIGURE 4:**
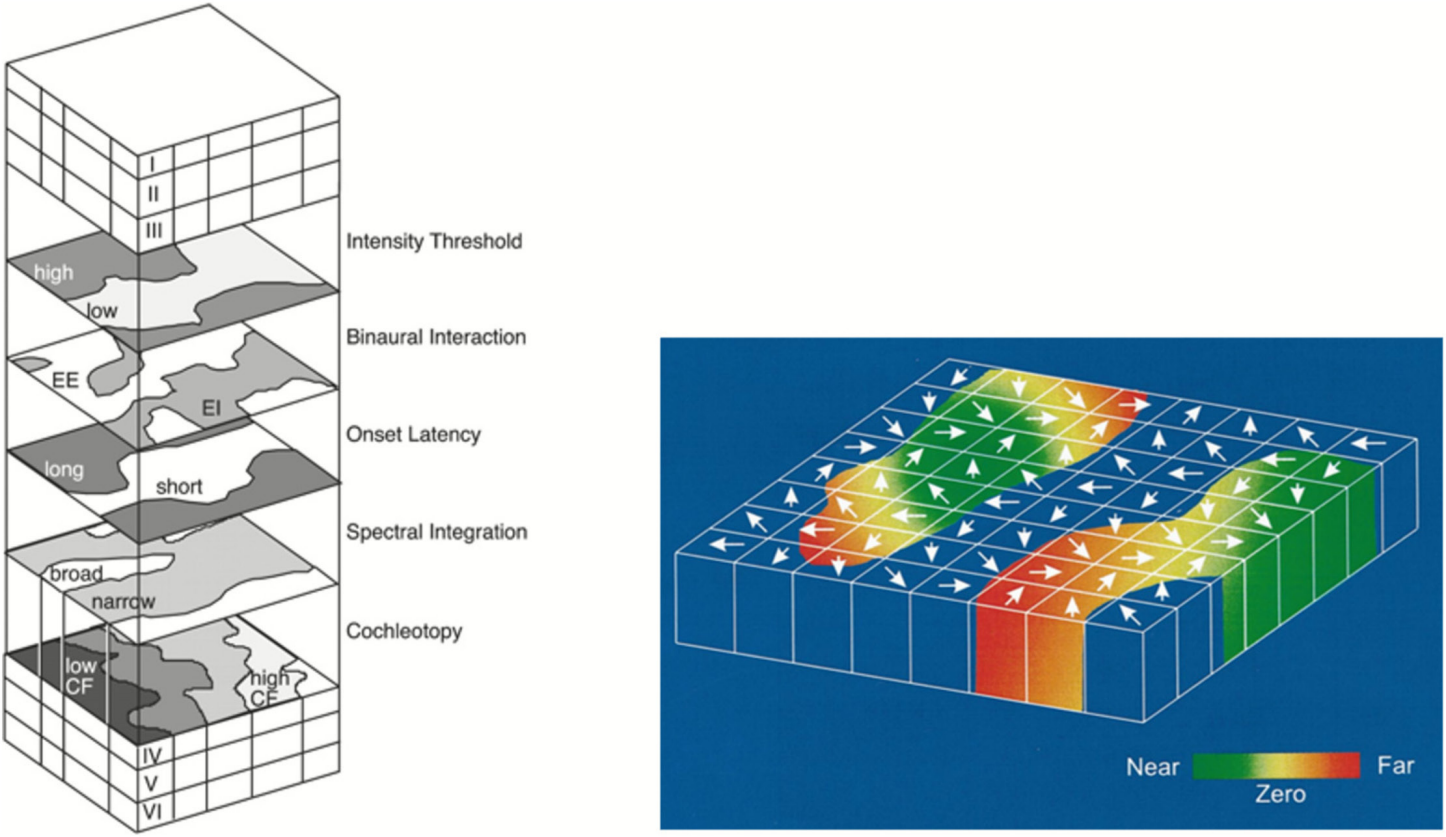
The problem of noncolumnar responses. *Left:* primary auditory cortex contains feature maps, but these do not extend across all cortical layers (Adapted from Linden and Schreiner 2003). *Right:* While all MT cells are orientation‐selective (white arrows), some are only weakly disparity selective (dark blue regions) Adapted from DeAngelis and Newsome (1999).

Second, the *problem of noncolumnar responses* is that while some areas are functionally organized in a columnar fashion (e.g., S1 and V1 in cats and macaques), many other cortical areas show noncolumnar responses. In primary auditory cortex, multiple feature maps exist but they are often layer‐specific (Figure 4, right). Thus, there seems to be no evidence for a uniformly sized auditory column (Swindale 1990). In visual area MT, direction‐selectivity does not change discretely and is arranged in a variable fashion. Additionally, disparity selective cells are distributed in a patchy noncolumnar manner over the direction‐selectivity map (Figure 4, left). This problem of noncolumnar response persists even when researchers use improved methods such as cytochrome oxidase staining (Horton and Adams 2005).

The third problem is the *problem of columnar inter‐ and intraspecies variation* (Barbas et al. 2022). Orientation columns are found in many mammals but are absent in rats and squirrels, even though these animals have cells that are highly orientation‐selective (Horton and Adams 2005). Racoons, beavers, and cats have whiskers but lack barrels, while guinea pigs have barrels but do not show whisking behavior (Horton and Adams 2005). Similarly, ocular dominance columns are present in some primates, but not others, while both have similar visual capacities (Purves et al. 1992). In squirrel monkeys, 30% of the cases examined lack ocular dominance structures (Horton and Adams 2005). In other individuals of the same species, only part of V1 exhibited ocular dominance columns, despite intact vision.

Taken together, the three problems suggest that “cortical column” fulfils the condition for retiring it from scientific practice. Because the three problems were not resolved by methodological improvements or more data, “cortical column” persistently fails its central epistemic goal: to identify an anatomically discrete, functionally modular building block that executes the same computation across areas and species (Haueis 2021). This epistemic goal shows that the three problems are not disconnected failures. The problem of missing boundaries questions that we can distinguish neocortical building blocks anatomically (da Costa and Martin 2010). The problem of noncolumnar responses questions whether columnarity is the only form of functional organization in neocortex (Catania 2002). The problem of species variation questions that columnar structure is universally present across mammalian species (Horton and Adams 2005). Together, the three problems also make it highly unlikely that columnar structures are species‐invariant units that compute the same function across varying inputs.

Note that the proposal to retire “cortical column” neither means that cortical columns do not exist, nor that “cortical column” cannot be used for other purposes. Rather, “cortical column” may refer to diverse kinds of cortical structure with varying functional significance, which are useful for other goals in current research, such as validating new experimental techniques (Yacoub et al. 2008; Pizzuti et al. 2023) or studying evolution and development of columnar structures (Barbas et al. 2022). None of these goals depends on using “cortical column” for the goal of identifying a cortical building block (Haueis 2021).

**FIGURE 5:**
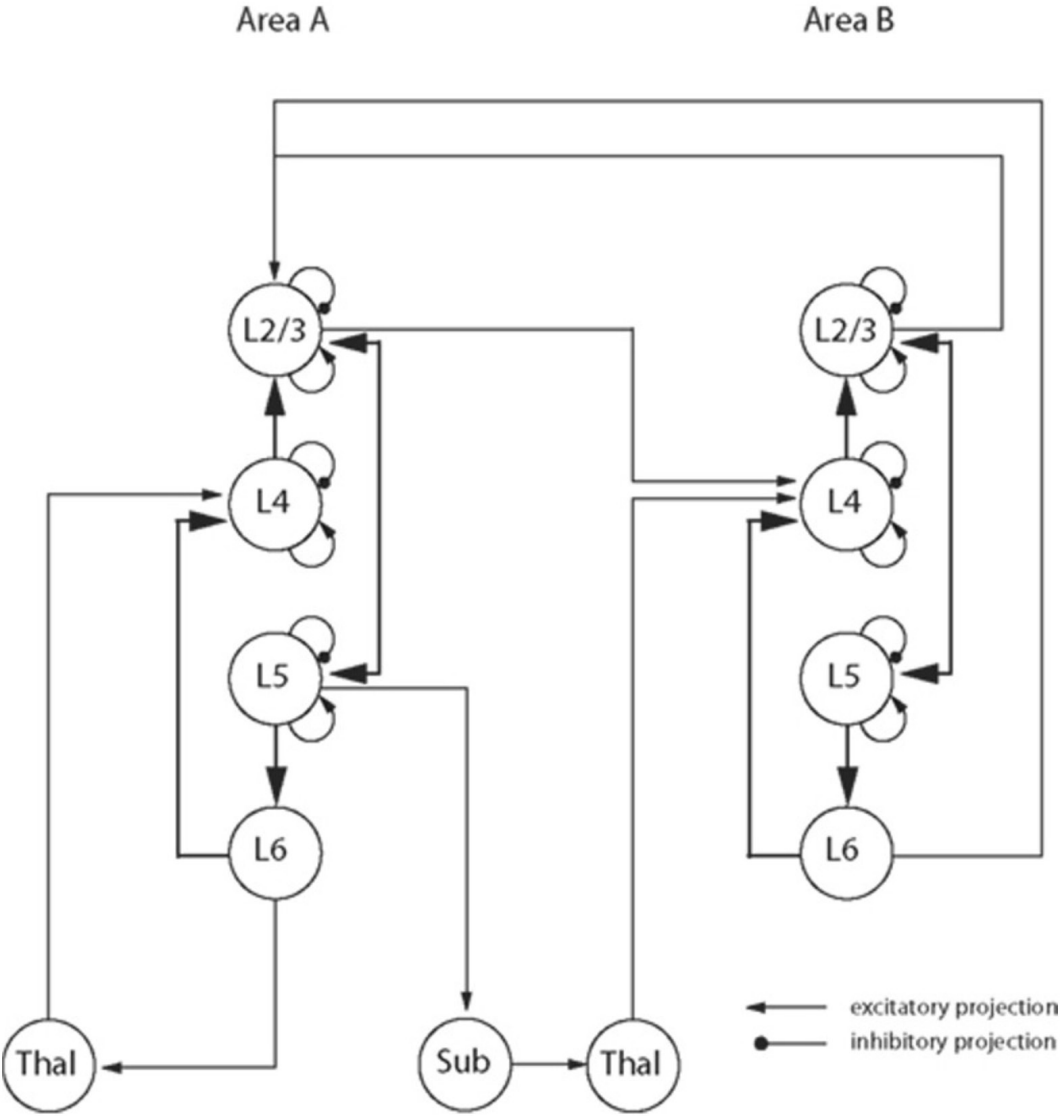
Wiring diagram of the canonical microcircuit, which is derived from intracellular recordings and horseradish peroxidase staining of neurons in cat V1. Inhibitory and excitatory L4 populations receive weak thalamic input, and send feedforward connections to L2/3 populations that are recurrently connected to L5 populations. L5 populations feedforward to L6 populations and subcortical areas. No spatial dimension is specified, different populations respond differently to stimuli, and the displayed wiring is assumed to be repeated across cortical areas and different species (Adapted from da Costa and Martin 2010).

We can now also turn to the question what concept should replace “cortical column” if we choose to continue our search for a cortical building block. A prominent proposal is “canonical microcircuit” (da Costa and Martin 2010). This concept can be used to identify a building block because it describes neocortical circuit organization in terms of stereotypical connection patterns between excitatory and inhibitory neuron types (Figure 5).

According to its proponents, “canonical microcircuit” contributes to this goal while avoiding the first and second problems that plague the column concept. First, “canonical microcircuit” avoids the problem of missing boundaries by representing circuit structure without specifying a discrete spatial boundary at the mesoscale, which does not exist in neocortex (Figure 3). Second, “canonical microcircuit” avoids the problem of noncolumnar responses by describing cells with functionally heterogenous response properties that do not all connect in a “like‐to‐like” manner (da Costa and Martin 2010). Regarding the problem of species variation, while some comparisons of circuit organization between cat, macaque, and rodent neocortex exist (Beul and Hilgetag 2015), it is currently an open question whether “canonical microcircuit” describes circuit organization that is universal across mammals.

Depending on whether we accept the conditions for retiring or replacing “cortical column,” we should change our understanding of neocortical circuit organization more or less drastically. If we reject both conditions, then we can continue to claim that “cortical column” identifies a neocortical building block. To do so, we would need to find ways to overcome the three problems, which have persisted over seven decades of column research. If we accept the first condition but reject the second, we can accept that “cortical column” refers to diverse kinds of structure but have to give up the goal of finding a neocortical building block at the mesoscale. Under this interpretation, “cortical column” remains helpful for other research goals. But when it comes to describing circuit organization, we should stop using it and should rather build area‐ and species‐specific descriptions of cortical circuits (Horton and Adams 2005; Haueis 2021). If we accept both conditions, then we should replace “cortical column” with “canonical microcircuit” and understand the neocortical building block as a nondiscrete infrastructure whose elements are dynamically recruited at any given moment (da Costa and Martin 2010).

## 4 How Developing Concepts Changes Our Understanding: The Case of “Hierarchy”

The success or failure of introducing, retiring, or replacing a concept depends on how well the concept is aligned with the epistemic goals of the research community. In this section, we refine this view in two ways. The first refinement is that the success and failure of concepts reflect their contributions to scientific understanding: successful concepts enable accurate descriptions, classifications, and explanations of phenomena, while flawed concepts reveal gaps in our understanding. The second refinement is that besides introducing and retiring/replacing concepts, conceptual development often requires negotiating multiple uses and interpretations of existing concepts.

This section brings both refinements together around the case of “hierarchy.” Illustrating our first refinement, “hierarchy” developed multiple meanings, such that researchers have currently various options of how to relate them when pursuing epistemic goals. We sketch three such options: *substantiation* refines entrenched ideas incrementally, *conflict* forces decisive theoretical shifts, and *pluralism* reconciles divergent interpretations by expanding explanatory scope. The options of relating the meanings of “hierarchy” illustrate our second refinement, since each reflects a different understanding of how the brain is hierarchically organized. Our discussion thus reveals that while epistemic goals help heuristically define success and failure, conceptual development impacts our understanding of brain organization in a piecemeal manner.

### 4.1 When Should We Revise a Concept to Enhance Neuroscientific Understanding?

The concept of hierarchy is central to how systems neuroscientists describe connectivity patterns, classify brain areas, and explain how these connected brain areas underlie behaviorally relevant information processing in the brain. Using “hierarchy” to pursue these interlocking descriptive, classificatory, and explanatory goals contributes to a better understanding of how the brain is organized. There are currently two uses of “hierarchy,” each of which contributes to these epistemic goals in a different manner. The first use comes with a representational meaning: Based on tract‐tracing data, it describes an anatomical hierarchy of feedforward, feedback, and lateral connections which underlies a sequence of input–output relations between brain areas (Felleman and Van Essen 1991). Based on electrophysiological recordings or functional imaging, it classifies areas within the hierarchy based on the specific types of information they represent (Hubel and Wiesel 1962). Based on this classification, the representational use of “hierarchy” then explains cognitive functions as the transformation of these specific information types from lower to higher hierarchical levels.

Consider the visual hierarchy: V4 is at a higher level than V1 because it represents color categories, whereas V1 represents only wavelength. A different part of V4 represents complex shapes rather than V1's representation of local orientation. Based on this classification, researchers can explain how a particular area contributes to a cognitive phenomenon (e.g., how V4 contributes to color perception), or how a hierarchy of areas underlies a whole range of phenomena (e.g., how the visual hierarchy underlies phenomena of visual processing). By aiding how we describe, classify, and explain structure–function relationships in the brain, this representational interpretation of “hierarchy” has greatly advanced our understanding of brain organization in sensory systems, motor systems, and association areas (Mesulam 1998; Grafton and Hamilton 2007; Badre et al. 2010).

The second, newer use of “hierarchy” comes with a topological meaning because it involves mathematical techniques and concepts from graph theory, alongside the aforementioned techniques. An area is at a higher hierarchical level if it is more *central*, i.e., if it has more widespread influence on the network of interconnected brain areas. Centrality can be measured differently, e.g., by calculating degree, betweenness centrality, or clustering coefficient, and it underlies graph‐theoretical descriptions of hubs and modules (van den Heuvel and Sporns 2013).

Crucially, we can use these measures and definitions to classify brain areas hierarchically without specifying the representational architecture of the brain. For example, da Costa and Sporns (2005) used degree and clustering coefficient to describe connectivity patterns in the macaque visual system. They used this description to classify areas hierarchically based on their influence on the rest of the network, without specifying “representational stages of streams.” Similarly, Sporns and Betzel (2016) used topological centrality to detect modules in “purely data driven way.” The same is true for dynamic measures. For example, the *integration value* of a node's activity is given by the number of other nodes to which it is functionally connected after the event that triggered the activity. Higher integration values thus signal higher influence on the network during the respective time period. One can use this measure to classify areas or networks into a hierarchy without specifying which type of information they represent (Deco and Kringelbach 2017). If this topological meaning of “hierarchy” is used to explain how the brain processes information, it may do so by specifying an area's information‐theoretic role in the network (e.g., broadcast, filter, and relay), rather than its representational content (Kötter and Stephan 2003).

### 4.2 Three Options to Revise “Hierarchy” in Systems Neuroscience

The existence of two meanings of “hierarchy” reflects a general phenomenon about developing concepts. When researchers extend a term to novel cases, e.g., by using new techniques or applying it to different scales, the term frequently acquires multiple meanings (Haueis 2024). A case in point is “gene” which biologists extended from intergenerational patterns to intracellular components, giving rise to Mendelian, classical, and several molecular meanings of “gene” (Brigandt 2010; Orgogozo et al. 2016). There are multiple options to relate these meanings, each with distinct implications of how we understand inheritance (Darden 2006; Baetu 2010; Rheinberger and Müller‐Wille 2018). In parallel, we show now that there are multiple options to relate the meanings of “hierarchy,” each with different implications of how we understand brain organization.

The first option is that the new, topological meaning of “hierarchy” adds information about brain organization that adds substance to, and is compatible with the old, representational meaning of “hierarchy.” What topological studies add is a more detailed picture of connectivity, but these details support interpreting “hierarchy” as representational and functional specificity. Evidence for this first option comes from studies showing that topological modularity analyses of anatomical connectivity align with well‐known functional divisions in the brain (Sporns et al. 2007; Hagmann et al. 2008; Meunier et al. 2009).

**FIGURE 6:**
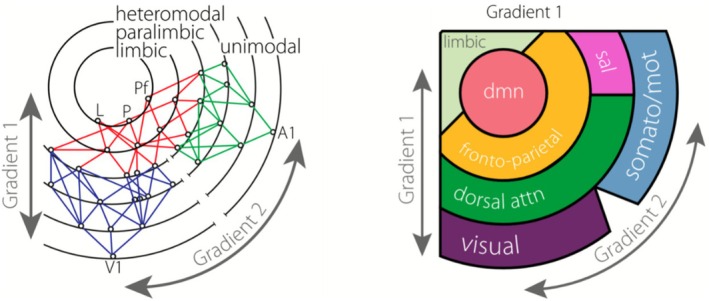
The Mesulam model (left) is extrapolated from macaque tract‐tracing, lesion and electrophysiology data, and orders areas in a hierarchy according to information represented in unimodal, heteromodal, and transmodal areas. The Margulies model is based on diffusion embedding of resting state functional connectivity data (right) and situates the DMN at the top of Mesulam's representational hierarchy. *Source:* Adapted from Margulies et al. (2016).

Another example of this is our own previous work, which uses diffusion map embedding to describe gradients of functional connectivity data (Section 3.1). There is an interpretation of the first gradient in terms of *representational abstraction*, which adds to, and is compatible with Mesulam's model of hierarchical brain organization (Mesulam 1998). It situates the DMN at the top of a known representational hierarchy that proceeds from unimodal sensory to transmodal association areas (Figure 6).

This first option implies that we should understand hierarchical brain organization mainly through the lens of the traditional framework developed by authors like Hubel and Wiesel (1977), Felleman and Van Essen (1991), and Mesulam (1998). Recent topological uses of “hierarchy” add details about connectivity and situate newly discovered entities like the DMN (Section 3) in this hierarchy. That is why Burnston and Haueis (2021) call this option “substantiation”: These novel studies add further substance to the understanding that hierarchical brain organization is about representation and functional specificity.

**FIGURE 7:**
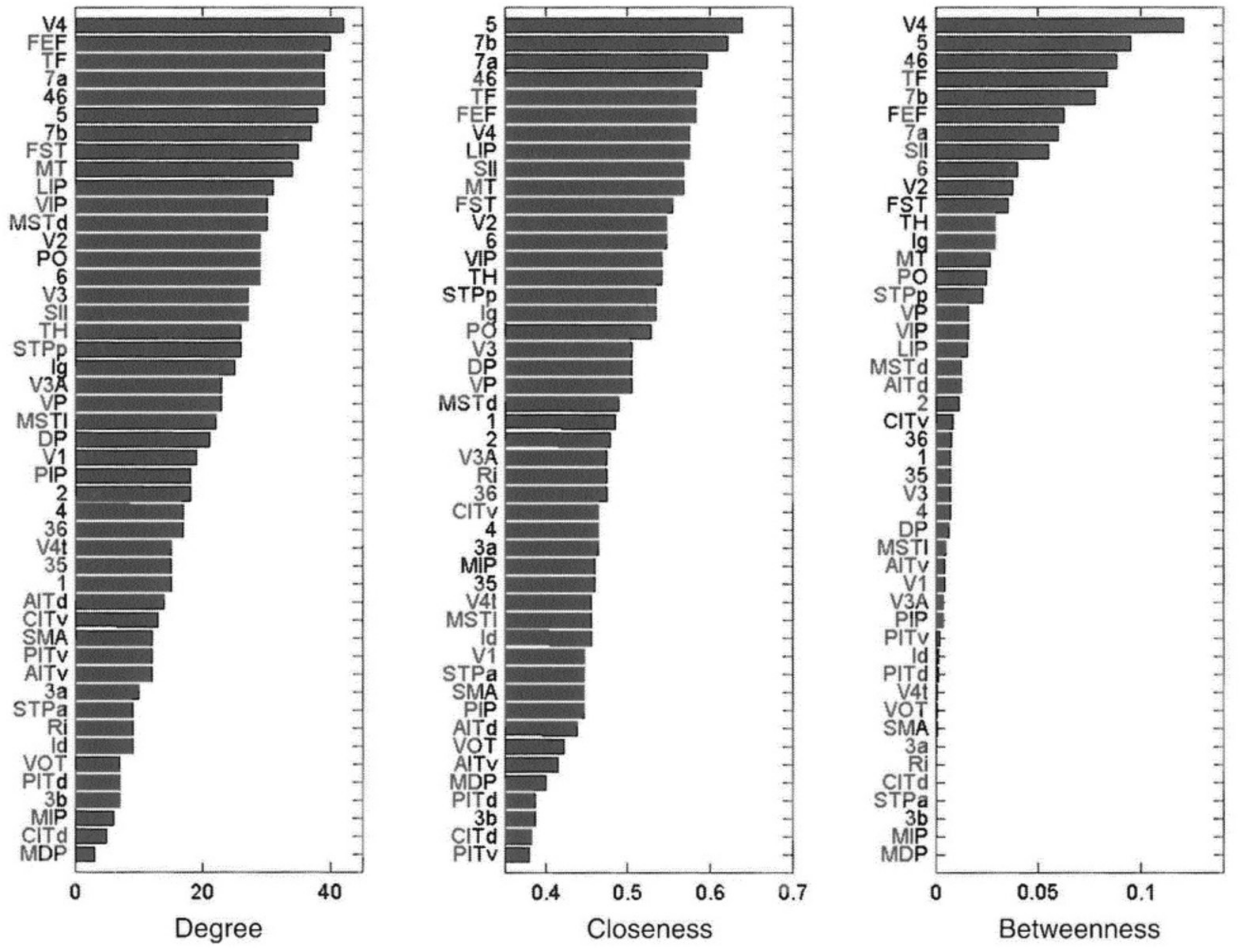
Measurements of centrality based on Macaque anatomical connectivity data. Degree denotes the percentage of actual out of all possible connections. Closeness centrality is defined as the reciprocal of the sum of the length of the shortest paths between the node and all other nodes in the graph. Betweenness centrality specifies how many shortest paths between any two nodes pass through the node of interest. V4 ranks highest for degree and betweenness centrality and ranks 7th for closeness centrality compared to all other brain areas (Adapted from Sporns 2011, fig. 2.9).

But not all cases of using “hierarchy” are of this kind. Sometimes the topological meaning of “hierarchy” *conflicts* with the representational meaning. This option gets its name from conflicting evidence about the hierarchical positions of brain areas or networks (Hegdé and Van Essen 2007, see Burnston and Haueis 2021 for details). Consider the example of V4, which the representational interpretation places in the middle of the visual hierarchy (Felleman and Van Essen 1991). By contrast, graph‐theoretical analyses of anatomical connectivity show that V4 has one of the highest centrality values in the entire brain, which is shown in Figure 7.

If we choose the conflict option, then we need to drastically change our understanding of brain organization. This option is similar to replacing “cortical column” with “canonical microcircuit.” There we saw two competing terms to pursue one epistemic goal (identifying the building block). Here, we have two meanings of the same term (“hierarchy”), which compete for three goals that together form part of our understanding of brain organization. Instead of talking about representation and abstraction of information, we should interpret “hierarchy” to connote the influence an area has on the brain, e.g., by broadcasting information more widely (Kötter and Stephan 2003; Deco and Kringelbach 2017). If we weigh conflicting evidence highly, then the topological meaning of “hierarchy” reflects an understanding of hierarchical brain organization that is incompatible with the representational meaning of this term.

Finally, the third option is that the two meanings “hierarchy” neither substantiate nor conflict but can coexist alongside one another. In philosophy, such positions are grouped under the umbrella of *pluralism*, which can itself take various forms (Ludwig and Ruphy 2024). In our case, there may actually be a plurality of distinct goals that researchers pursue when using “hierarchy.” The representational meaning of “hierarchy” contributes to explain how signals are in fact processed by the brain, whereas the topological meaning helps us describe the efficiency of communication under constraints like minimizing wiring length (Van Den Heuvel and Sporns 2011). The different uses of “hierarchy” can coexist because each helps us to achieve a distinct epistemic goal. It could also be the case that the brain itself has plural forms of hierarchical organization, each of which are elucidated by the representational and topological meanings of “hierarchy.” In contexts where organisms make specific perceptual judgments, hierarchical organization is well described as one of representational specificity. But in contexts of action and deliberation, where evaluative and motivational influences are crucial, more complicated forms of signal processing that are mediated by topological properties may be required (Burnston and Haueis 2021).

Choosing a form of pluralism changes our understanding of brain organization in ways that lie between substantiation and conflict. If there is a plurality of goals, the representational meaning could continue to play a role for certain goals, while others require switching to the topological meaning of “hierarchy.” If there are plural forms of organization, the traditional representational picture accurately describes one of these forms, while they are better captured as implementing a topological hierarchy. But when choosing this option, researchers should stop talking as if there is *one* hierarchy in the brain, independently of context (Honey et al. 2007).

The case of “hierarchy” illustrates the general tendency of concepts to acquire new meanings when researchers develop new measures and models and expand them into unknown territory (Bloch‐Mullins 2020; Haueis 2024). These new meanings may change our understanding of the relevant phenomena, depending on how they relate to already established uses of the concept. If a new meaning adds substance to the old one, much of our existing understanding can remain intact, since only further details are added. If the meanings conflict, we may have to drastically change our understanding. We cannot simultaneously maintain conflicting interpretations of the same concept but need to decide—at least eventually—which one to adopt. Finally, pluralist options suggest a change of understanding that lies between substantiation and conflict. Unlike substantiation, the novel use reveals that the existing understanding is rather incomplete, because the old use either does not contribute to important epistemic goals, or because it does not include important phenomena in the investigated domain.

The fact that there can be evidence for each kind of option also underscores the main point of our paper. While the strength of evidence for each option may vary, the success or failure of adopting an option also crucially depends on epistemic goals. Choosing the substantiation option is called for if the established interpretation of a concept already leads to mostly accurate descriptions of patterns in the data, provides useful classifications of these patterns, and allows adequate explanations of the relevant phenomena. Choosing conflict calls the epistemic success of the old approach into question. Replacing the old with a new meaning is likely successful if the new approach (here: topological analyses of hierarchy) can solve outstanding conceptual issues (Haueis and Kästner 2022) or foster further technological and methodological developments (Favela 2022). Choosing pluralism consequently argues for a divide‐and‐conquer approach, where distinct interpretations of a concept contribute to distinct goals, each of which contributes to a better understanding of the relevant phenomena.

In sum, this section refined the goal‐based view of conceptual development to show how neuroscientists can balance the preservation of legacy frameworks with the demand for innovation. We thus underscore that conceptual development is not a binary outcome but a spectrum of epistemic negotiation, where methodological advances both constrain and liberate our understanding of the brain.

## 5 Conclusion

The evolution of concepts in neuroscience, whether by introducing new terms, extending and revising existing ones, or retiring outdated frameworks, is a fundamentally goal‐driven process. The primary lesson of our paper is thus that we should make our epistemic goals explicit to better evaluate the success and failure of concepts in neuroscience. Yet, we also emphasize that pursuing epistemic goals such as description, classification, explanation, or prediction depends on a collaborative dialogue between experimentalists, theorists, and modelers (Favela 2022). This is a dialogue that can be fostered by a philosophical toolkit that emphasizes the dynamics of conceptual development. In so doing, it helps us avoid conceptual entrenchment and prioritize frameworks that best address the epistemic goals of neuroscience.

The case studies we have presented underscore another critical lesson: Conceptual success depends on balancing continuity with adaptability (Haueis 2023, 2024). When novel evidence challenges existing frameworks, we must decide whether to refine, replace, or integrate interpretations. This flexibility in developing concepts is vital for tackling the brain's multiscale complexity, where no single perspective suffices. The conceptual journey from grandmother cell to sparse coding (Barwich 2019), or from cortical column to canonical microcircuit, illustrates that progress lies not in declaring concepts “right” or “wrong,” but in iteratively revising them to capture our evolving understanding of the brain.

## Author Contributions

Philipp Haueis and Daniel S. Margulies equally contributed to the conceptualization of the article. Philipp Haueis wrote the original draft, and both authors revised the manuscript.

## Funding

This project was supported in part by funding to D.S.M. from the European Research Council (ERC) under the European Union's Horizon 2020 Research and Innovation Programme (grant agreement No. 866533‐CORTIGRAD).

## Conflicts of Interest

The authors declare no conflicts of interest.

## Data Availability

No original data were analyzed for this article.

## Endnotes

^1^
The use of “hypothesis” in articles does not reliably indicate whether a study is exploratory or confirmatory. Researchers may use this term for presentational purposes even when their study used exploratory research strategies (Rowbottom and Alexander 2012). This use of “hypothesis” usually marks a relation between two features of the system the study investigated (Earman and Salmon 1992, 1999), rather than hypotheses derived from a theoretical model of the system under study (Giere et al. 2006). Exploratory experiments in neuroscience are hypothesis‐free in the latter but not the former sense.

^2^
Focusing on mechanisms, Haueis (2023) restricts this first condition to causal properties. Here we expand to capture that neuroscientific concepts can also be anchored in noncausal features such as quantitative measures of topography or brain topology (see Sections 2.2 and 4.1, e.g.,).

^3^
Philosophers frequently distinguish between the term in language and the corresponding concept (e.g., as a mental representation) by writing, e.g., “default mode network” and DEFAULT MODE NETWORK. As a result, the epistemic impact of linguistic phenomena like strategic ambiguity can be easily missed.
